# EuMicroSat*db*: A database for microsatellites in the sequenced genomes of eukaryotes

**DOI:** 10.1186/1471-2164-8-225

**Published:** 2007-07-10

**Authors:** Veenu Aishwarya, Atul Grover, Prakash C Sharma

**Affiliations:** 1 University School of Biotechnology, Guru Gobind Singh Indraprastha University, Kashmere Gate, Delhi 110 006, India; 2 Shri Radha Kissen Kanoria Center for Advanced Studies in Bioscience and Biotechnology, Banasthali Vidyapith, Banasthali 304 022 (Raj.) India

## Abstract

**Background:**

Microsatellites have immense utility as molecular markers in different fields like genome characterization and mapping, phylogeny and evolutionary biology. Existing microsatellite databases are of limited utility for experimental and computational biologists with regard to their content and information output. EuMicroSat*db *(**Eu**karyotic **MicroSat**ellite ***d***ata***b***ase)  is a web based relational database for easy and efficient positional mining of microsatellites from sequenced eukaryotic genomes.

**Description:**

A user friendly web interface has been developed for microsatellite data retrieval using Active Server Pages (ASP). The backend database codes for data extraction and assembly have been written using Perl based scripts and C++. Precise need based microsatellites data retrieval is possible using different input parameters like microsatellite type (simple perfect or compound perfect), repeat unit length (mono- to hexa-nucleotide), repeat number, microsatellite length and chromosomal location in the genome. Furthermore, information about clustering of different microsatellites in the genome can also be retrieved. Finally, to facilitate primer designing for PCR amplification of any desired microsatellite locus, 200 bp upstream and downstream sequences are provided.

**Conclusion:**

The database allows easy systematic retrieval of comprehensive information about simple and compound microsatellites, microsatellite clusters and their locus coordinates in 31 sequenced eukaryotic genomes. The information content of the database is useful in different areas of research like gene tagging, genome mapping, population genetics, germplasm characterization and in understanding microsatellite dynamics in eukaryotic genomes.

## Background

Microsatellites, also called as simple sequence repeats (SSRs) or simple tandem repeats (STRs) are ubiquitous component of eukaryotic genomes. A microsatellite consists of a specific sequence of DNA which contains 1–6 bp long (mono- to hexa- nucleotide) tandem repeats viz. (A)_16_, (GA)_20_, (GATA)_30_. Over the years, molecular biologists have increasingly exploited these sequences for diverse applications.

With the whole genome sequencing initiatives of various eukaryotic organisms, large amount of genomic sequence data has accumulated over the last few years. These sequence resources available in the public domain have also served as an attractive source of *in silico *mining of microsatellite sequences [1-5]. *In silico *mining of these sequences offers advantage in terms of time, labour and cost over conventional isolation from genomic libraries. Similarly, ESTs have also been screened for the presence of microsatellites [6-8]. However, finding potentially useful microsatellites occupying specific genomic regions still remains a challenge for the molecular biologists. Availability of this information can facilitate molecular mapping of desired traits and preparation of linkage maps saturated with evenly distributed SSR markers.

Popularity of *in silico *mining methods has led to the construction of various microsatellite databases in recent years, each with a different emphasis. For instance, MICdb [9] provides information on microsatellites spanning coding and non-coding regions, their frequency, size and repeat sequence. A recent version of this database covers 19 archeal, 155 eubacterial and 287 viral genomes. SilkSatDb [10] incorporates microsatellites extracted from the available ESTs and genomic sequences of the silkmoth (*Bombyx mori*). This database also stores data on polymorphism status of different microsatellite loci. Similarly, mouse genomic microsatellites are collected in the Mouse Microsatellite Database of Japan (MMDBJ) [11]. CMD (Cotton Microsatellite Database) is a web-based relational database providing centralized access to publicly available cotton microsatellites. The database also provides a useful resource for mapping and related data pertaining to major cotton microsatellite projects [12]. Satellog [13] database catalogues triplet repeats associated with human disorders. Similarly Microsat2006 [14] database catalogues human microsatellite repeats. Taiwanese Polymorphic Microsatellite Database (TPMD) provides data on microsatellite mapping in the Taiwanese populations [15]. Molecular Mycology Research Laboratory, Westmead, Australia has created an SSR database [16] that stores information on microsatellite repeats in nine fungal genomes. InSatDb [17] provides size, type (perfect and compound) and location (intron, exon, upstream or transposons) of microsatellites in five insect genomes. Some other databases [18,19], although published earlier are currently inaccessible. In conclusion, existing microsatellite databases either are very specific in their content and application or have limited utility to a wider audience. Thus, a collection of whole genome eukaryotic microsatellite data at a single platform is still not available. Recognizing this gap, we have developed a comprehensive database for easy retrieval of information on microsatellites distributed in the sequenced eukaryotic genomes. The database named as EuMicroSat*db *(**Eu**karyotic **MicroSat**ellite ***d***ata***b***ase) presents a web-based user friendly interface for the extraction of both simple and compound microsatellites from 31 eukaryotic genomes assembled as chromosomes. Important features of this database are compared with those of existing databases in Table 1.

**Table 1:** Comparison of various microsatellite databases, available in the public domain

**Database**	**Details on**					**Coverage**
		
	**Simple Repeats**	**Compound Repeats**	**Clustering information**	**Genomic Positions**	**Flanking Sequences**	
MICdb [9]	Y	Y	N	Y	Y	19 archeal, 155 bacterial and 287 viral genomes
SilkSatDb [10]	Y	Y	N	N	Y	Silkworm
MMDBJ [11]	Y	Y	N	N	N	Mouse
CMD [12]	Y	Y	N	N	Y	Cotton
Satellog [13]	Y	N	N	Y	N	Human
Database of Molecular Mycology Research Lab. [16]	Y	N	N	Y	N	9 fungal genomes
InSatDb [17]	Y	Y	N	Y	Y	5 insect genomes
MRD [18]	Y	N	N	N	N	8 eukaryotic genomes
SSRD [19]	Y	N	N	N	N	Human
EuMicroSat*db*	Y	Y	Y	Y	Y	31 eukaryotic genomes

## Construction and Content

**Figure 1:**
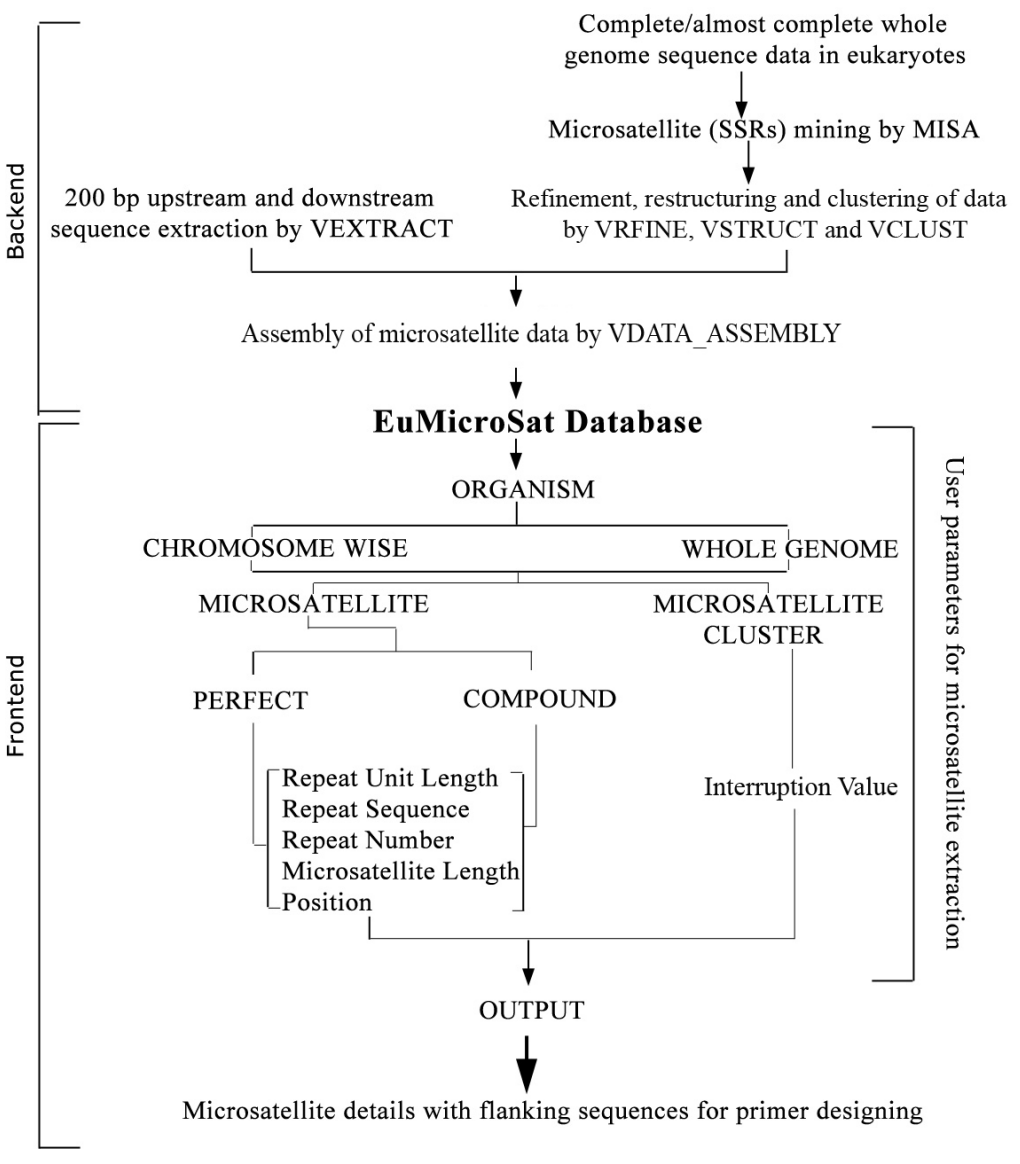
**Construction scheme of EuMicroSat*db***. Figure describing methodology used for preparation of backend (codes used in the preparation of files in the database) and front end (the web interface of the database).

**Figure 2:**
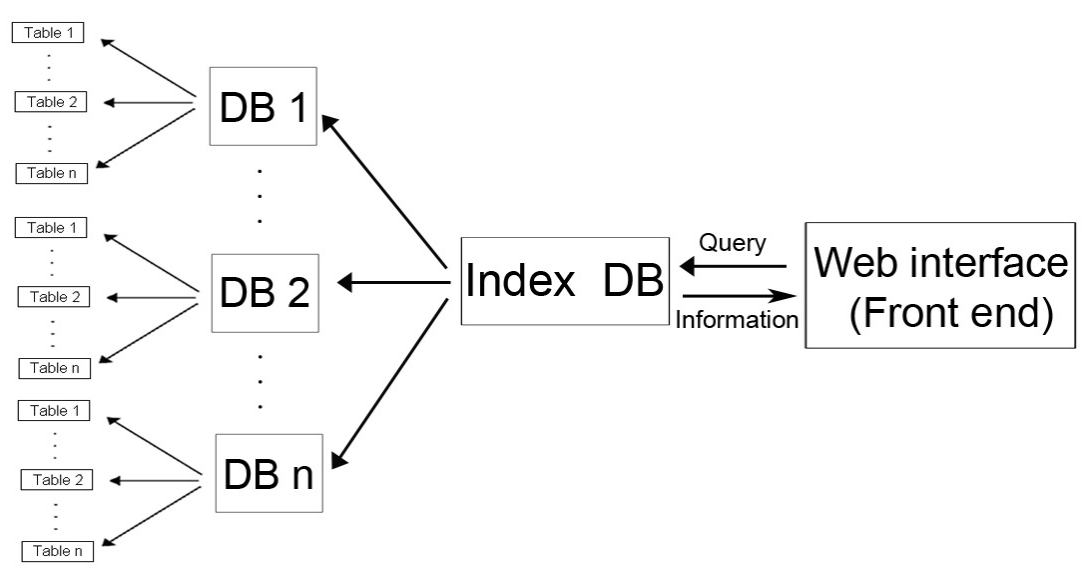
**Architecture of EuMicroSat*db***. Scheme of EuMicroSat*db *describing the outline of the database.

EuMicroSat*db *is a platform independent relational database. The basic scheme followed for the development of EuMicroSat*db *involved following steps: (1) whole genome sequences were downloaded from various sources like Ensembl [20], The National Center for Biotechnology Information (NCBI) [21], Genolevures 2 [22], International Rice Genome Sequencing Project (IRGSP) [23], Beijing Genomics Institute (BGI) [24], The Arabidopsis Information Resource (TAIR) [25] (Table 2) and scanned using a simple sequence repeat mining tool called MISA [26]; (2) filtering and restructuring of the required data using novel algorithms, **VRFINE **(creates "rfine" file that has sequence information of microsatellites) and **VRSTRUCT **(uses "rfine" file and processes it to make three separate files for repeat number, repeat motif and repeat unit length); (3) extraction of 200 bp upstream and downstream flanking sequences using a C++ program called **VEXTRACT **that creates two separate files, one each for upstream and downstream sequences; (4) microsatellite clustering information was generated using a Perl based script **VCLUST**, and (5) all the data generated by above algorithms were reassembled into a data file using another Perl based script **VDATA_ASSEMBL**. This file was then imported in MS-ACCESS as a table. The overall scheme of database construction is explained in figure 1. Sub-databases were constructed for individual genomes by importing all data files as tables, each representing one chromosome. Finally, an Index-database was created that communicates with these sub-databases. Front end web interface was developed using ASP that communicates with the Index database for data retrieval. The overall architecture of the database is outlined in figure 2.

**Table 2:** Details of genomes included in EuMicroSat*db*

**Species**	**Build/Assembly/Ver**.	**Source**	**Web Link**
*Saccharomyces cerevisiae*	SGD 1, Nov 2005	Ensembl	
*Schizosaccharomyces pombe*	NCBI release	NCBI	
*Aspergillus oryzae *RIB40	NCBI release	NCBI	
*Aspergillus fumigatus*	NCBI release	NCBI	
*Cryptococcus neoformans *var*JEC21*	NCBI release	NCBI	
*Encephalitozoon cuniculi*	NCBI release	NCBI	
*Eremothecium gossypii*	NCBI release	NCBI	
*Candida glabrata *CBS138	Genolevures 2 Release 2, May 2006	Genolevures	
*Debaryomyces hansenii*	Genolevures 2 Release 2, May 2006	Genolevures	
*Kluyveromyces lactis*	Genolevures 2 Release 2, May 2006	Genolevures	
*Yarrowia lipolytica*	Genolevures 2 Release 2, May 2006	Genolevures	
*Caenorhabditis elegans*	WS160, July 2006	Ensembl	
*Plasmodium falciparum*	NCBI release	NCBI	
*Anopheles gambiae*	AgamP3, Feb 2006	Ensembl	
*Drosophila melanogaster*	BDGP 4.3, July 2005	Ensembl	
*Apis mellifera*	NCBI release	NCBI	
*Tribolium castaneum*	NCBI release	NCBI	
*Oryza sativa *ssp.*japonica*	Build 4.0	IRGSP	
*Oryza sativa *ssp. *indica*	2003-08-01 BGI	BGI	
*Arabidopsis thaliana*	ver. Jan 22 2004	TAIR	
*Ciona intestinalis*	JGI 2, Mar 2005	Ensembl	
*Tetraodon nigroviridis*	TETRAODON 7, Apr 2003	Ensembl	
*Danio rerio*	Zv6, Mar 2006	Ensembl	
*Rattus norvegicus*	RGSC 3.4, Dec 2004	Ensembl	
*Mus musculus*	NCBI m36, Dec 2005	Ensembl	
*Gallus gallus*	WASHU2, May 2006	Ensembl	
*Canis familiaris*	CanFam 1.0, July 2004	Ensembl	
*Macaca mulatta*	MMUL 1.0, Feb 2006	Ensembl	
*Bos taurus*	Btau_3.1, Aug 2006	Ensembl	
*Pan troglodytes*	PanTro 2.1, Mar 2006	Ensembl	
*Homo sapiens*	NCBI 36, Oct 2005	Ensembl	

## Utility and Discussion

The EuMicroSat*db *allows mining of different microsatellites along with their physical location on chromosomes in completely/almost completely sequenced eukaryotic genomes. At present, the database has over 10 million entries of microsatellites covering 31 genomes (Table 2). More genomes will be included in the database as and when their whole genome sequences are published and made available in the public domain.

**Figure 3:**
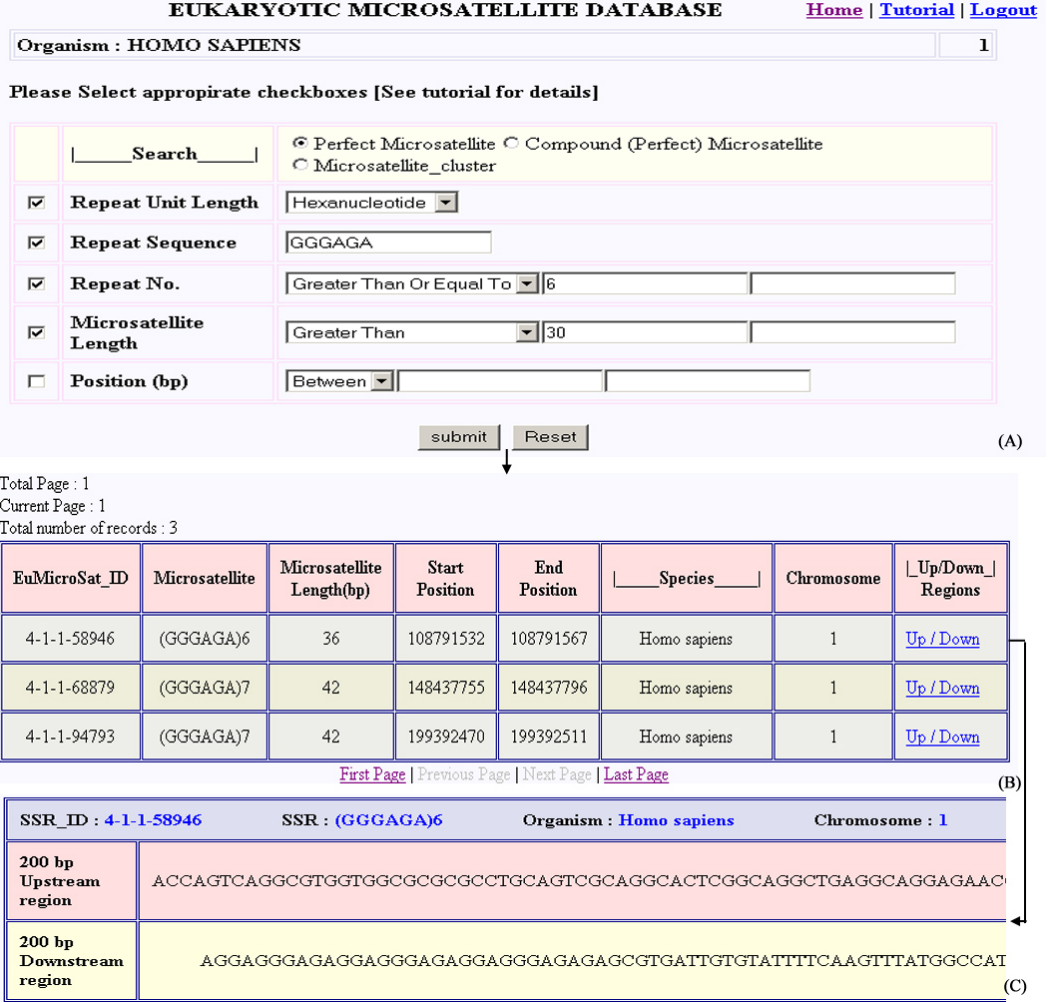
**Web interface for simple microsatellite searching**. Web interface showing (A) various input parameters used for simple microsatellite based search, (B) output of the query, and (C) 200 bp upstream/downstream sequences for a particular microsatellite.

User can search for perfect repeats, compound (perfect) repeats and microsatellite clusters. EuMicroSat*db *database can be searched using following need based input parameters: Repeat unit length: the basic unit that is tandemly repeated in the microsatellite ranging from mononucleotide to hexanucleotide; Repeat sequence: this parameter allows the user to search microsatellite for a specific base sequence, for example, AT, GCG, etc.; Repeat number: is used to search microsatellites on the basis of repeat number of the microsatellite e.g. (CCT)_9 _has a repeat number of 9, (AGAGG)_10 _has a repeat number of 10; Microsatellite length: searches microsatellites on the basis of their total length in base pairs e.g. (TTGCA)_5 _has a length of 25 bp; Position: defined locations on the chromosome in terms of base pairs can be specified; Microsatellite cluster: search can also be performed to look for adjacent microsatellites. Further, if the user wants to design primers for PCR amplification of the desired microsatellite locus, the database also provides 200 bp upstream and downstream regions of all the microsatellite loci. The search options are further explained with the help of some case studies given in a power point tutorial available on the database website. Figure 3 displays the user interface for searching microsatellites by using one or more of the above-mentioned basic parameters.

**Figure 4:**
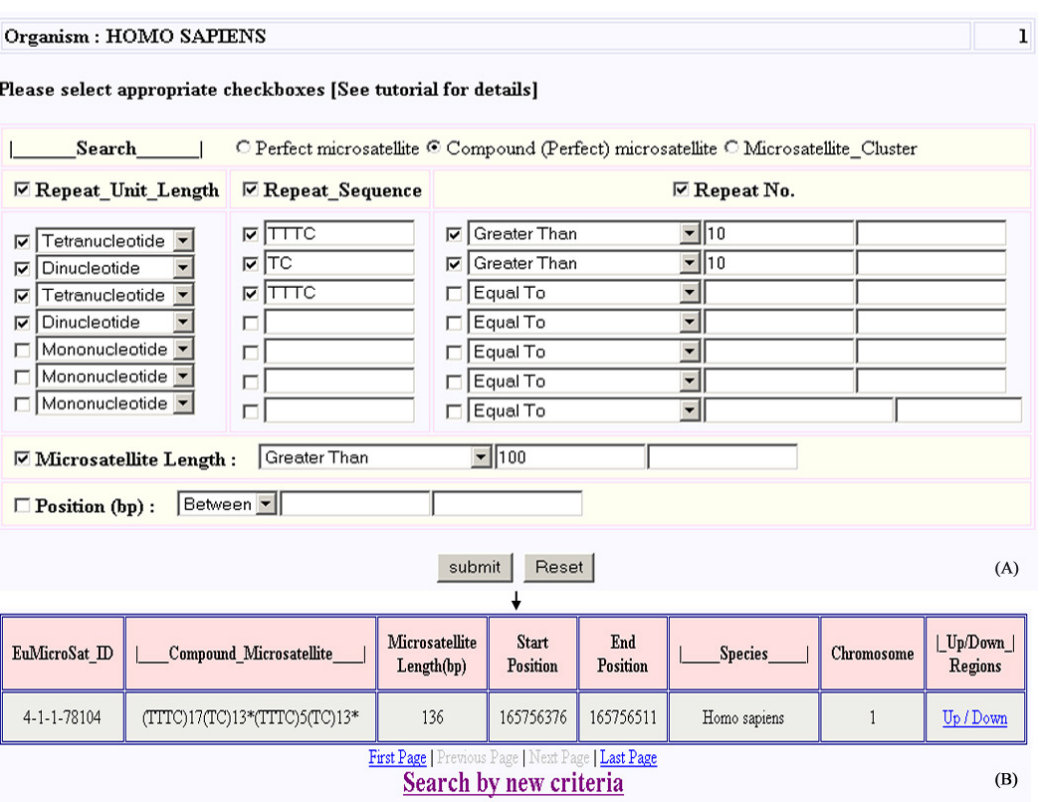
**Web interface for searching compound microsatellite**. Web interface showing (A) various input parameters used for searching compound microsatellite, and (B) output of the query.

The unique feature of this database is the extraction of both simple and compound microsatellites. Compound repeats can be searched by specifying motifs desired in the combination. For example, if a user wants to search for a compound microsatellite from chromosome 1 of *Homo sapiens *which is more than 100 bp in length, has a TTTC-TC-TTTC repeat combination with the fourth association being a dinucleotide repeat, with repeat number greater than 10 for TTTC and TC, search can be made by using the parameters specified in figure 4A. The output of this query is shown in figure 4B.

User can identify the genomic regions showing high microsatellite density to study microsatellite clustering in the genome [27] by defining the size of interruption between neighbouring microsatellites as explained in the database tutorial.

EuMicroSat*db *is likely to be adopted as a useful tool to study the relative occurrence and distribution of microsatellites across eukaryotic genomes. The information may have diverse applications. The user can extract the position of the microsatellite on the chromosome and thus can link it with the gene co-ordinates, which are available on various public domains. In this way, microsatellites located in the vicinity of genes may be identified. These microsatellites will hopefully prove to be more useful for gene tagging and to investigate the role of microsatellites in gene regulation. Similarly, microsatellites spanning desired genomic regions can be selected and used for further saturation of existing molecular maps. Since microsatellite show varying levels of cross amplification among related genomes, microsatellites from the genomes included in the present database can be exploited for developing markers in the related species where sufficient STMS markers are still not available. The compound microsatellites being hypervariable can prove to be a potential source of highly polymorphic markers.

Incorporation of multiple sub-databases in EuMicroSat*db *ensures faster exchange of information and unlimited expansion of the database. The database will be upgraded regularly as and when draft assemblies are updated and new genomes are sequenced. EuMicroSat*db *is compatible with multi-user environment. The efficiency of data retrieval is maintained during simultaneous access by many users.

## Conclusion

EuMicrosat*db *has been developed for genome wide mining of microsatellite in 31 completely sequenced eukaryotic genomes considering the immense utility of these sequences for a variety of experiments. Various parameters have been carefully inducted to allow comprehensive search of simple and compound microsatellites and to identify microsatellite clusters across the genomes. Links to retrieve flanking sequences (200 bp upstream and downstream) are provided to design primers for PCR amplification of desired motifs. EuMicroSat*db *will provide a useful resource for mining microsatellites to be used in gene tagging, comparative genomics and genetic diversity based studies in different genomes

## Availability and requirements

EuMicroSat*db *is a platform independent relational database publicly available at 

## Authors' contributions

VA was mainly responsible for writing codes, designing the architecture of the database and execution of the study. AG participated in designing of the database and helped in the preparation of the manuscript. PCS conceived, coordinated and supervised the study. All authors read and approved the final manuscript.
